# Simplified ZrTiO_*x*_-based RRAM cell structure with rectifying characteristics by integrating Ni/n + -Si diode

**DOI:** 10.1186/1556-276X-9-275

**Published:** 2014-05-30

**Authors:** Chia-Chun Lin, Yung-Hsien Wu, You-Tai Chang, Cherng-En Sun

**Affiliations:** 1 Department of Engineering and System Science, National Tsing Hua University, Hsinchu 30013, Taiwan

**Keywords:** 1D1R, Metal/semiconductor, Schottky diode, RRAM, Rectifying behavior, ZrTiO_*x*_, Retention, Endurance

## Abstract

A simplified one-diode one-resistor (1D1R) resistive switching memory cell that uses only four layers of TaN/ZrTiO_*x*_/Ni/n^+^-Si was proposed to suppress sneak current where TaN/ZrTiO_*x*_/Ni can be regarded as a resistive-switching random access memory (RRAM) device while Ni/n^+^-Si acts as an Schottky diode. This is the first RRAM cell structure that employs metal/semiconductor Schottky diode for current rectifying. The 1D1R cell exhibits bipolar switching behavior with SET/RESET voltage close to 1 V without requiring a forming process. More importantly, the cell shows tight resistance distribution for different states, significantly rectifying characteristics with forward/reverse current ratio higher than 10^3^ and a resistance ratio larger than 10^3^ between two states. Furthermore, the cell also displays desirable reliability performance in terms of long data retention time of up to 10^4^ s and robust endurance of 10^5^ cycles. Based on the promising characteristics, the four-layer 1D1R structure holds the great potential for next-generation nonvolatile memory technology.

## Background

As conventional flash memory is approaching its scaling limits, resistive-switching random access memory (RRAM), one of the most promising emerging nonvolatile memories, holds the potential to replace it for future memory-hungry applications because of superior speed, higher density, and complementary metal-oxide-semiconductor (CMOS) compatibility [1-4]. For the last decade, although many dielectrics have shown resistive switching characteristics, undesirable cross-talk through neighboring cells due to sneak current leads to read disturbance and limits the array size. To circumvent the issue, series connection of one diode (1D) with one RRAM (1R) to form the so-called 1D1R cell has been proposed since the sneak current can be suppressed by the rectifying the characteristics without sacrificing the storage density. The requirements of the diode include large ratio between forward and reverse current (*F*/*R* ratio) under read operation, fab-friendly process, and many types of diodes were discussed in the literature. Metal-insulator-metal (MIM)-based diodes such as Pt/TiO_2_/Ti [5,6], Pt/CoO/IZO/Pt [7], and Pt/TiO_*x*_/Pt [8] meet the requirement of high *F*/*R* ratio, however, the implementation of these diodes necessitates at least three layers and the adoption of high-work function Pt, increasing the complexity of integration and process cost respectively. Besides aforementioned diodes, W/TiO_*x*_/Ni-based MIM diode [9] is promising since it achieves *F*/*R* ratio larger than 1,000 without using Pt and successfully demonstrates the integration with bipolar RRAM. Nevertheless, three layers are still required to implement the diodes. Other types of diode include p-type/n-type oxide-based diodes such as NiO_*x*_/TiO_*x*_[10], CuO_*x*_/InZnO_*x*_[11], and NiO_*x*_/ITO_*x*_[12], or polymer film such as P3HT/n-ZnO [13]. Even though high *F*/*R* ratio is achieved, most oxides are not compatible with incumbent ultra large scale integration (ULSI) technology. Diode based on p-type/n-type Si is another viable technology; although it has been integrated with phase change memory [14], related research on RRAM has not been reported. In addition, with top and bottom electrodes, these diodes require four layers to be implemented; thus, the issue of process complexity still remains. By integrating the aforementioned diodes with RRAM devices, process that needs more than four layers is indispensable.

Recently, without the need of a diode, RRAM devices with self-rectifying behavior have been widely developed because of the simpler process. For self-rectifying RRAM devices, dielectric and electrode should be carefully selected to concurrently meet the requirement of large *F*/*R* ratio for diode and high *R*_HRS_/*R*_LRS_ ratio for RRAM where *R*_HRS_ and *R*_LRS_ respectively denote the resistance at high-resistance state (HRS) and low-resistance state (LRS). Most device structures with self-rectifying behavior such as Cu/a-Si/WO_3_/Pt [15], Pt/Al/PCMO/Pt [16], and Pt/ZrO_*x*_/HfO_*x*_/TiN/HfO_*x*_/ZrO_*x*_/Pt [17] still possess unsatisfactory *R*_HRS_/*R*_LRS_ ratio (approximately 10) and *F*/*R* ratio (approximately 100). In addition, it usually requires at least four layers to implement self-rectifying characteristics for aforementioned RRAM devices and the structure compromises the advantage of simple process of self-rectifying devices. Ni/AlO_*x*_/n^+^-Si [18], a simpler structure with self-rectifying characteristics, exhibits desirable *R*_HRS_/*R*_LRS_ and *F*/*R* ratio. However, forming voltage larger than 5 V is required, and there is room to improve the operation voltage which is higher than 2 V. In this work, a novel 1D1R cell structure based on TaN/ZrTiO_*x*_/Ni/n^+^-Si was proposed where TaN/ZrTiO_*x*_/Ni was employed as the resistive switching element and Ni/n^+^-Si played the role of Schottky diode. The reason to adopt ZrTiO_*x*_ is that it has been shown to have desirable RRAM characteristics [19]. Compared to those published in the literature, the intriguing points of this work lie in four aspects: (1) This is the first structure that uses metal/semiconductor Schottky diodes to rectify current characteristics and the whole structure requires only four layers which are much simpler than other 1D1R structures and even comparable to self-rectifying devices. (2) This 1D1R cell displays desirable electrical characteristics in terms of forming-free property, *R*_HRS_/*R*_LRS_ ratio higher than 10^3^, *F*/*R* ratio larger than 10^3^, operation voltage close to 1 V, negligible resistance change up to 10^4^ s retention time at 125°C, and robust endurance of 10^5^ cycles. (3) Unlike some 1D1R structures that use special materials as diode, all the layers used in this work are fab-friendly and can be fully integrated with existing ULSI process.

## Methods

N-type Si wafer with doping concentration of 2 × 10^17^ cm^−3^ was used as the starting material for 1D1R cell fabrication. A 35-nm Ni was initially deposited on the Si wafer as the bottom electrode of MIM-based RRAM device. Note that the Ni layer on the n-type Si substrate also formed the Schottky diode because of the metal/semiconductor junction. Next, a 10-nm oxygen-deficient ZrTiO_*x*_ film was deposited by e-beam evaporation from a pre-mixed source that contains ZrO_2_ and Ti at room temperature as the resistance switching dielectric. TaN of 35 nm was then deposited and patterned by shadow mask as the top electrode. Finally, complete 1D1R cells with the structure of TaN/ZrTiO_*x*_/Ni/n^+^-Si were formed. For electrical characterization, voltage was applied on the top electrode with the grounded Si substrate. Separate RRAM (TaN/ZrTiO_*x*_/Ni) and Schottky diode (Ni/n^+^-Si) were also formed to evaluate the behavior of single device. Note that single RRAM devices were fabricated on SiO_2_ rather than Si substrate for better isolation so that pure RRAM performance can be measured. All the electrical data were measured by devices with the area of 250 μm × 250 μm. In addition to electrical analysis, transmission electron microscopy (TEM) and x-ray diffraction (XRD) were respectively used to characterize the interface property between Ni/n^+^-Si and to study the crystallinity of the switching dielectric ZrTiO_*x*_.

## Results and discussion

### Physical analysis of 1D and 1R structure

**Figure 1:**
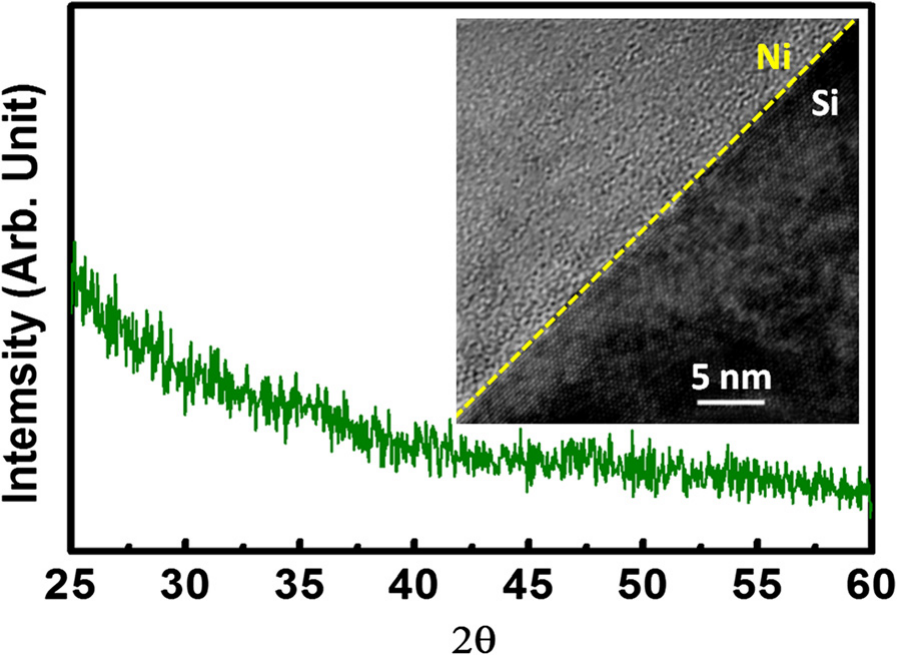
**XRD pattern for ZrTiO**_***x ***_**dielectric used in 1D1R cell.** The inset shows the cross-sectional TEM for Ni/n^+^-Si interface.

Figure 1 shows the XRD spectrum for ZrTiO_*x*_ film prior to the deposition of top electrode TaN. No diffraction peaks are observed and it implies that the film is amorphous phase. In fact, it has been reported that ZrTiO_4_ can be crystallized in orthorhombic phase at 600°C [20]. Without any thermal treatment in this work, it is reasonable for the ZrTiO_*x*_ film to be amorphous. The inset shows the cross-sectional TEM image for the interface between Ni and n^+^-Si. Besides the clear single-crystal Si structure, the Ni film is found to be amorphous without observing any crystalline layer near Si interface. This phenomenon suggests that no nickel silicide was formed in the device since the formation of nickel silicide will result in crystalline layer. Nickel silicide is a commonly used material to improve contact resistance and has been well studied in the literature [21] from which Ni_2_Si, NiSi, and NiSi_2_ can be respectively formed at 250°C, 350°C, and 700°C. Again, since no thermal treatment was employed in this work, the Ni film of amorphous phase without forming any silicide is expected.

### DC behavior for 1D, 1R, and 1D1R devices

**Figure 2:**
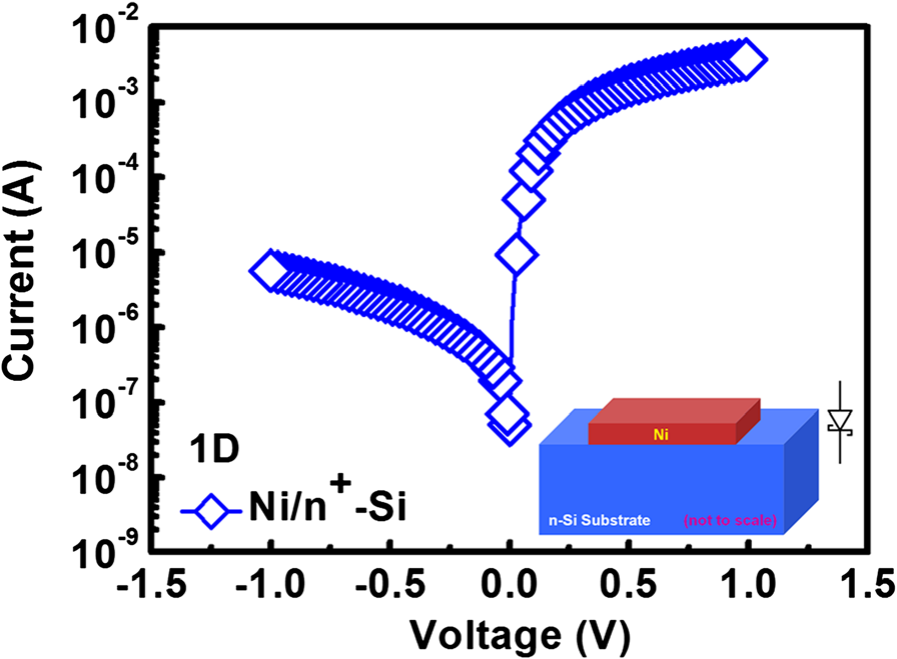
***I*-*V *** **curve for Ni/n**^**+**^**-Si based 1D cell.**

**Figure 3:**
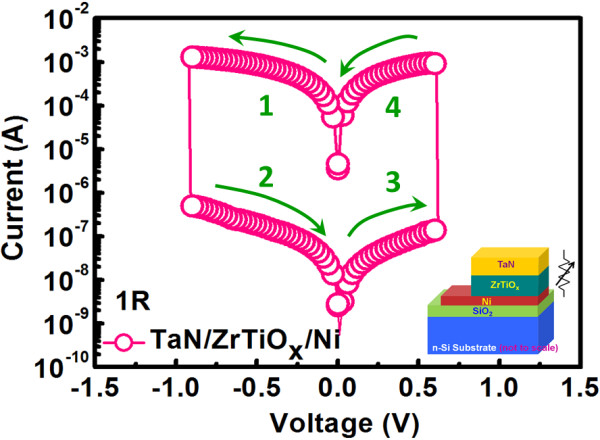
***I*-*V *** **hysteresis curve for TaN/ZrTiO**_***x***_**/Ni based 1R cell.**

**Figure 4:**
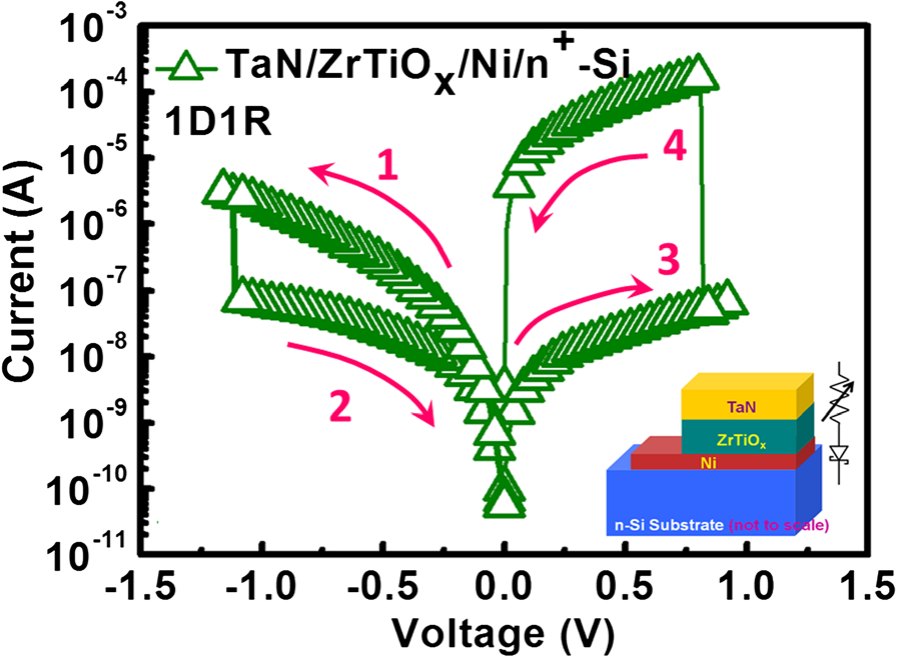
***I*-*V *** **hysteresis curve for TaN/ZrTiO**_***x***_**/Ni/n**^**+**^**-Si based 1D1R cell.**

**Figure 5:**
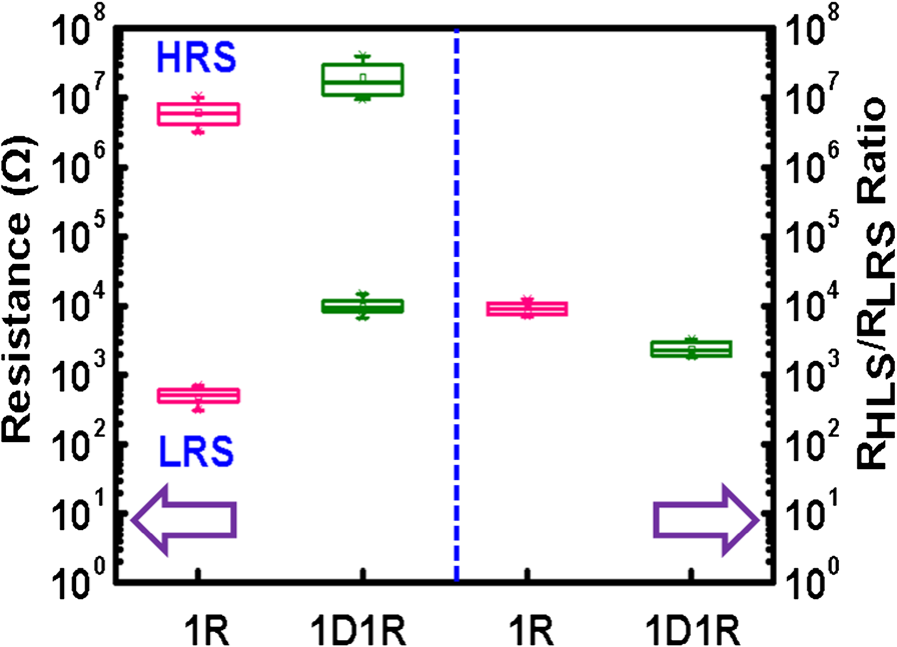
**Statistical distribution of resistance and *R***_**HRS**_**/*R***_**LRS **_**ratio for 1R and 1D1R cells.**

Figure 2 shows the current-voltage (*I*-*V*) curves for Ni/n^+^-Si based diode and it was measured with grounded n^+^-Si, and a typical Schottky diode curve is demonstrated because of the metal/semiconductor junction. The *F*/*R* ratio for this diode measured at ±0.2 V is about 10^3^ which proves good rectifying properties. In fact, from the exponential forward bias region, the barrier height for Ni/n^+^-Si junction is extracted to be 0.66 eV with the consideration of image force-lowering effect. To further enhance the *F*/*R* ratio, the doping concentration of Si can be modulated to be lower so that the effect of image force lowering and tunneling can be suppressed. Figure 3 shows the switching behavior for TaN/ZrTiO_*x*_/Ni-based RRAM devices and it demonstrates self-compliance, forming-free characteristics with SET/RESET voltage lower than 1 V, and *R*_HRS_/*R*_LRS_ ratio of 9 × 10^3^ at read voltage of +0.1 V. The initial LRS can be ascribed to the existence of a pre-existed filament that is composed of oxygen vacancies in the nonstoichiometric ZrTiO_*x*_. As a negative bias is applied on the top electrode TaN (positive bias applied on bottom electrode Ni), it will build an electric field that drives oxygen vacancies to move toward the top electrode TaN and therefore the filament will be ruptured, making devices switch to HRS. In fact, the voltage-driven oxygen vacancies movement has been proposed in the literature as the switching mechanism for other dielectrics [22,23]. On the other hand, applying a positive bias on the top electrode TaN (negative bias applied on bottom electrode) under HRS would repel the oxygen vacancies near the top electrode toward the bottom electrode and re-align the oxygen vacancies to form conducting filaments because of the downward electric field, switching devices from HRS to LRS. In addition, the current conduction mechanism at HRS and LRS is respectively extracted to be Schottky emission and ohmic conduction (not shown). Because the current conduction mechanism at LRS is extracted to be ohmic conduction, the LRS current at both polarities is similar. Since individual diode and RRAM have shown good electrical properties, the performance of device formed by stacking RRAM and diode (TaN/ZrTiO_*x*_/Ni/n^+^-Si) was analyzed and the hysteresis *I*-*V* curve is shown in Figure 4. The stacked device (1D1R) still represents resistive switching behavior. Represented in Figure 5 is the statistical distribution of resistance and *R*_HRS_/*R*_LRS_ ratio for 1R and 1D1R devices. Even with the integration of a diode, the resistance distribution does not degrade and the tight distribution is advantageous for cell integration. The major differences from 1R cell are summarized as follows:

1. The RESET current decreases to be around 10^−5^ A which is two orders lower than that of 1R cell. This improvement mainly comes from the connected reverse-biased diode which limits the current flowing through it. The phenomenon is similar to other 1D1R structure reported in [9,10].

2. The current level at LRS demonstrates significant rectifying characteristics for both polarities. At ±0.1 V, the F/R ratio can be up to 10^3^, which resulted from the series connection of the diode and capable of suppressing the sneak current effect.

3. The operation current becomes lower while *R*_HRS_/*R*_LRS_ ratio degrades to approximately 2,300 at +0.1 V. Nevertheless, the ratio is still large enough to distinguish logic ‘1’ and ‘0’. The lower current level can be explained by the fact that for a given applied voltage, there is voltage drop on the diode, and therefore the effective voltage drop on the RRAM is smaller than that of 1R cell. In addition, for positive bias which corresponds to diode operated under forward region because the effective voltage drop on the RRAM directly depends on its resistance state and the nonlinear *I*-*V* characteristics of the diode, the *R*_HRS_/*R*_LRS_ ratio becomes degraded.

4. SET/RESET voltage slightly increases. This is attributed to voltage drop across the diode and therefore a larger voltage is required to form equivalent voltage on the RRAM. Nevertheless, the SET/RESET voltage is still close to 1 V which is beneficial for low-power operation.

### Conduction mechanism and retention characteristics

**Figure 6:**
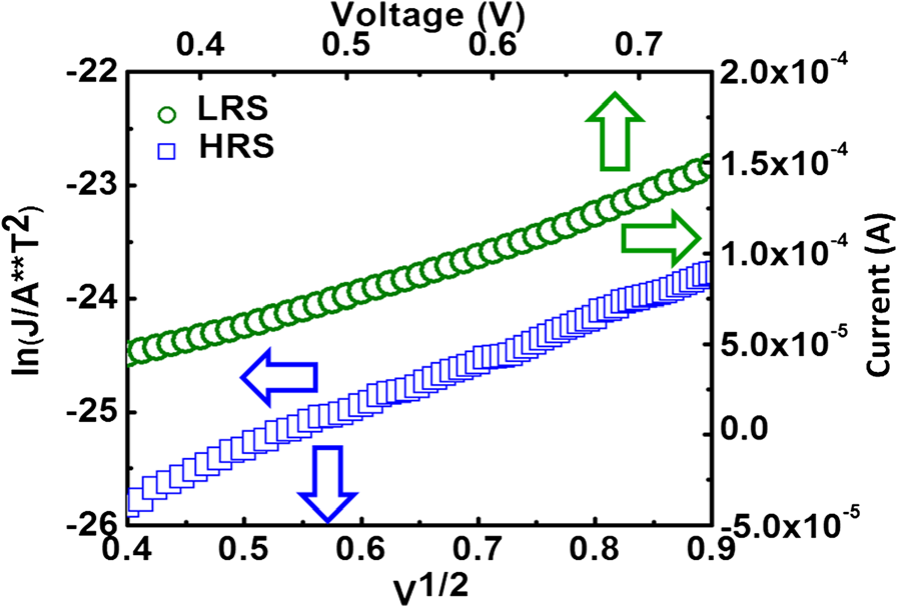
**Current conduction mechanism at HRS and LRS for TaN/ZrTiO**_***x***_**/Ni/n**^**+**^**-Si-based 1D1R cell.**

**Figure 7:**
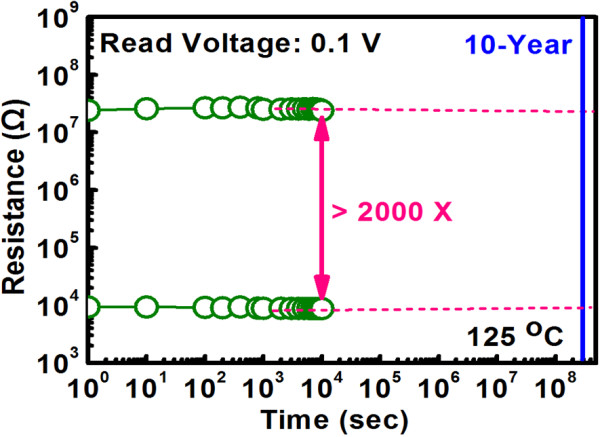
**Retention characteristic measured at 125°C for TaN/ZrTiO**_***x***_**/Ni/n**^**+**^**-Si based 1D1R cell.**

**Figure 8:**
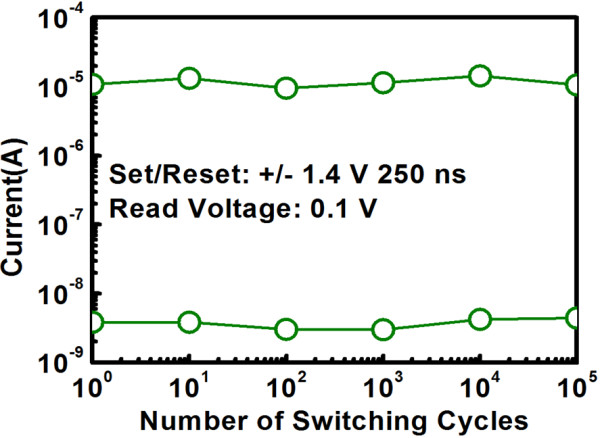
Endurance performance measured by applying continuous ±1.4 V pulse trains of 250 ns for 1D1R cell.

Figure 6 explores the conduction mechanism for LRS and HRS at positive bias by analyzing the correlation between current and voltage for 1D1R cell. The same as the case of 1R cell, for positive bias, it can be found that ohmic conduction and Schottky emission correspond to LRS and HRS respectively. The reason why conduction mechanism remains unchanged can be explained as follows. Under positive bias, the Schottky diode operates in forward region. For LRS, a relatively large voltage drop across the diode is expected, and the fully conducting diode can be regarded as the series connection of an ideal diode with cut-in voltage *V*_D0_ and a dynamic resistor (*r*_d_), according to piecewise linear diode model. Based on this model, the ohmic conduction for LRS is reasonable since there are two resistors (from RRAM and diode) connected in series in the equivalent circuit. On the other hand, for HRS, the voltage drop across the diode is small which may make its operating point less than the cut-in voltage and therefore the conduction mechanism for the diode is dominated by Schottky emission. Combined with the Schottky emission conduction for single RRAM at HRS, the same conduction mechanism is expected for 1D1R cell. To assess the ability to maintain the stored data for 1D1R cell, retention performance was measured at 125°C with a read voltage of 0.1 V and the result is shown in Figure 7 which demonstrates *R*_HRS_/*R*_LRS_ ratio over 2,000 with negligible degradation up to 10^4^ s. Figure 8 shows the switching endurance for 1D1R cell by applying continuous ±1.4 V pulse of 250 ns and the current was read at 0.1 V. The sensing margin can achieve 2,286 times initially and then slightly degrade to 2,105 times after 10^5^ cycles. This stable endurance performance implies that the 1D1R cell is robust enough to be used for practical memory applications.

## Conclusions

A simplified 1D1R cell with only four layers was proposed by adopting TaN/ZrTiO_*x*_/Ni/n^+^-Si structure. Table 1[8,10,15,16,24] summarizes the main device characteristics of this work, and other RRAM structures with rectifying properties are also listed for comparison. The 1D1R cell developed in this work shows promising characteristics in terms of low operation voltage close to 1 V, tight resistance distribution for different states, large *F*/*R* ratio of 10^3^, high *R*_HRS_/*R*_LRS_ ratio of approximately 2,300, long retention time up to 10^4^ s, and robust endurance up to 10^5^ cycles, which are beneficial for lower power consumption, sneak current suppression, and data storage. Further optimization of the diode process is required to enhance rectifying performance which could further suppress the sneak current and make a larger array size possible.

**Table 1:** Comparison of main device characteristics for RRAM devices with rectifying property

**RRAM structure**	**Diode**	**R**_**HRS**_**/R**_**LRS **_**ratio**	**Set voltage (V)**	**Reset voltage (V)**	**F/R ratio (V)**
Pt/TiO_*x*_/Pt [8]	Pt/TiO_*x*_/Pt	~10^2^ @ 1 V	~4.5 V	~2	<10^2^ @ ±0.5
Pt/NiO/Pt [10]	Pt/p-NiO_*x*_/n-TiO_*x*_/Pt	~10^3^		~ −3	10^5^ @ ±3
Pt/WO_3_/a-Si/Cu [15]	Self-rectified	~10^2^ @ 1 V	~1 V	~ −1.5	10^2^ @ ±1
Pt/A1/PCMO/Pt [16]	Self-rectified	10 @ 1 V			10 @ 4
NiSi/HfO_*x*_/TiN [24]	Self-rectified	>10^3^		~1.8	>10^3^ @ ±1
This work TaN/ZrTiO_*x*_/Ni	Ni/n^+^-Si	~2,300 @ 0.1 V	~0.75 V	~ −1	~10^3^ @ ±0.2

## Competing interests

The authors declare that they have no competing interests.

## Authors’ contributions

CCL made contributions to analysis and interpretation of data. YHW conceived of the study, participated in the coordination, and involved in drafting the manuscript. YTC designed and set up the experimental procedure, fabricated the devices, and acquired the data. CES conducted the electrical measurement of the devices. All authors read and approved the final manuscript.
